# Supplementary material for: Urban areas promotes shifts in the proportion of prey consumed by four raptor species (Accipitridae) in Mexico
Source: PeerJ. 2025 Oct 29;13:e20307. doi: 10.7717/peerj.20307 (PMC12579481; doi:10.7717/peerj.20307)
Supplement: Supplemental Information 1 — All types of prey reported for each species of raptor are indicated. The ranges in the table include data from males and females. The references from which the data were obtained are indicated in parentheses: a = Schnell 1994; b = Wheeler & Clark 1995; c = Fergusson-Lees Christie, 2005; d = Cartron et al., 2010; e = Duffy, 2012; f = Garay & Marín, 2019; g = Bibles et al., 2020; h = Stewart et al., 2023; i = Bierregaard et al., 2024; j = Johnson & Schnell, 2024; k = Rosenfield et al., 2024. [file peerj-13-20307-s001.docx]

| Supplemental Information 1. The morphological traits of the studied raptors and their prey types. All types of prey reported for each species of raptor are indicated. The ranges in the table include data from males and females. The references from which the data were obtained are indicated in parentheses: a =Schnell 1994; b = Wheeler & Clark 1995; c = Fergusson-Lees Christie 2005; d = Cartron et al. 2010; e =Duffy et al. 2012; f =Garay & Marín 2019; g = Bibles et al. 2020; h = Stewart et al. 2023; i = Bierregaard et al. 2024; j = Johnson & Schnell 2024; k = Rosenfield et al. 2024. | | | | | | |
| --- | --- | --- | --- | --- | --- | --- |
| Raptor species | Sexually dimorphic plumage | Age-related polymorphic plumage | Body length range (cm) | Wingspan range (cm) | Mass range (g) | Prey |
| Cooper’s Hawk | No (k) | Yes (k) | 37-47  (b,d) | 73-84  (b,d) | 341-528 (b,d) | Birds and, mammals (k) |
| Common Black Hawk | No (a,j) | Yes (a,j) | 43-56 (e,j) | 102-127 (e,j) | 793-1199 (j) | fish, crustaceans, lizards, snakes and mammals (j) |
| Roadside Hawk | No (i) | Yes (i) | 31-42 (c,f,i) | 64-92 (c) | 250-350 (f,i) | insects, spiders, reptiles, mammals, and birds (i) |
| Gray Hawk | No (g) | Yes (g) | 37-46 (h,g) | 75-94 (c) | 429-628 (g) | Lizards, snakes, birds and mammals (g) |
