# Supplementary material for: Urban areas promotes shifts in the proportion of prey consumed by four raptor species (Accipitridae) in Mexico
Source: PeerJ. 2025 Oct 29;13:e20307. doi: 10.7717/peerj.20307 (PMC12579481; doi:10.7717/peerj.20307)
Supplement: Supplemental Information 3 — Records were compiled through two sources: citizen science platforms (CS) and a review of published articles (L). Records associated with lowercase letters were obtained from the following publications: a = Hiraldo et al., 1991; b = Ibarra-Zimbrón et al., 2001; c = Mikula et al., 2015; d = Rodríguez-Canseco et al., 2015; e = Nahuat-Cervera et al., 2020; f = Ortega-Álvarez et al., 2022; g = Bello-Sánchez et al. 2021; h = Valencia-Herveth et al., 2022; i = Sánchez et al., 2023; and j = Escamilla-Cortés & García-Grajales, 2024. [file peerj-13-20307-s003.docx]

| Supplemental Information 4. Prey consumption records of four species of raptors in Mexico. Records were compiled through two sources: citizen science platforms (CS) and a review of published articles (L). Records associated with lowercase letters were obtained from the following publications: a = Hiraldo et al. 1991; b = Ibarra-Zimbrón et al., 2001; c = Mikula et al., 2015; d = Rodríguez-Canseco et al., 2015; e = Nahuat-Cervera et al., 2020; f = Ortega-Álvarez et al., 2022; g = Bello-Sánchez et al. 2021; h = Valencia-Herveth et al., 2022; i = Sánchez et al., 2023; and j = Escamilla-Cortés & García-Grajales 2024. | | | | | | | | |
| --- | --- | --- | --- | --- | --- | --- | --- | --- |
|  | **Cooper's Hawk** | | **Common Black Hawk** | | **Roadside Hawk** | | **Gray Hawk** | |
|  | CS | L | CS | L | CS | L | CS | L |
| **Myriapoda** | 0 | 0 | 0 | 0 | 0 | 0 | 0 | 0 |
| *Hemiscolopendra marginata* | 0 | 0 | 0 | 0 | 1 | 0 | 0 | 0 |
| **Decapoda** | 0 | 0 | 26 | 0 | 1 | 0 | 1 | 0 |
| *Procambarus clarkii* | 0 | 0 | 2 | 0 | 0 | 0 | 0 | 0 |
| *Cardisoma guanhumi* | 0 | 0 | 1 | 0 | 0 | 0 | 0 | 0 |
| *Ocypode occidentalis* | 0 | 0 | 1 | 0 | 0 | 0 | 0 | 0 |
| *Cardisoma crassum* | 0 | 0 | 0 | 1j | 0 | 0 | 0 | 0 |
| **Hexapoda** | 0 | 0 | 0 | 0 | 5 | 0 | 0 | 0 |
| Odonata | 0 | 0 | 1 | 0 | 1 | 0 | 0 | 0 |
| Dermaptera | 0 | 0 | 0 | 0 | 1 | 0 | 0 | 0 |
| *Stilpnochlora sp* | 0 | 0 | 1 | 0 | 0 | 0 | 0 | 0 |
| Hemiptera | 0 | 0 | 0 | 0 | 1 | 0 | 0 | 0 |
| **Actinopterygii** | 0 | 0 | 44 | 0 | 0 | 0 | 0 | 0 |
| Tetraodontidae | 0 | 0 | 1 | 0 | 0 | 0 | 0 | 0 |
| *Moxostoma austrinum* | 0 | 0 | 0 | 1a | 0 | 0 | 0 | 0 |
| **Amphibia** | 0 | 0 | 0 | 0 | 0 | 0 | 0 | 0 |
| Anura | 0 | 0 | 8 | 0 | 5 | 0 | 2 | 0 |
| **Mammalia** | 1 | 0 | 0 | 0 | 0 | 0 | 0 | 0 |
| Rodentia | 1 | 0 | 1 | 0 | 6 | 0 | 6 | 0 |
| *Otospermophilus variegatus* | 2 | 0 | 0 | 0 | 0 | 0 | 1 | 0 |
| Chiroptera | 0 | 1c | 0 | 0 | 0 | 1c | 0 | 1c |
| *Didelphis virginiana* | 0 | 0 | 0 | 0 | 0 | 0 | 2 | 0 |
| *Didelphis spp* | 0 | 0 | 0 | 0 | 0 | 0 | 1 | 0 |
| Lagomorpha | 0 | 0 | 0 | 0 | 1 | 0 | 0 | 0 |
| *Sylvilagus floridanus* | 0 | 1a | 0 | 0 | 0 | 0 | 0 | 0 |
| *Sigmodon leucotis* | 0 | 1a | 0 | 0 | 0 | 0 | 0 | 0 |
| *Sciurus aureogaster* | 0 | 0 | 0 | 0 | 0 | 0 | 1 | 0 |
| *Neotamias bulleri* | 0 | 1a | 0 | 0 | 0 | 0 | 0 | 0 |
| *Heterogeomys sp* | 0 | 0 | 0 | 0 | 0 | 0 | 1 | 0 |
| *Canis lupus familiaris* | 1 | 0 | 0 | 0 | 0 | 0 | 0 | 0 |
| **Reptilia** | 0 | 0 | 0 | 0 | 1 | 0 | 0 | 0 |
| Iguanidae | 0 | 0 | 0 | 0 | 13 | 0 | 12 | 0 |
| Lacertidae | 0 | 0 | 2 | 0 | 9 | 0 | 5 | 0 |
| Serpentes | 1 | 0 | 9 | 0 | 5 | 0 | 0 | 0 |
| Colubridae | 0 | 0 | 0 | 0 | 6 | 0 | 1 | 0 |
| *Aspidoscelis sp* | 1 | 0 | 0 | 0 | 3 | 0 | 1 | 0 |
| Aspidoscelis guttatus | 0 | 0 | 0 | 0 | 0 | 1g | 0 | 0 |
| Testudines | 0 | 0 | 4 | 0 | 0 | 0 | 0 | 0 |
| *Sceloporus sp* | 0 | 1a | 0 | 1a | 2 | 0 | 0 | 0 |
| *Holcosus undulatus* | 0 | 0 | 0 | 0 | 0 | 0 | 0 | 1e, 1h |
| *Thamnophis proximus* | 0 | 0 | 1 | 0 | 0 | 1e | 0 | 0 |
| *Lampropeltis polizona* | 0 | 1d | 0 | 0 | 0 | 0 | 0 | 1d |
| *Ameiva* | 0 | 0 | 0 | 0 | 1 | 0 | 0 | 0 |
| Scincidae | 0 | 0 | 0 | 0 | 1 | 0 | 0 | 0 |
| *Sceloporus poinsetti* | 0 | 1a | 0 | 0 | 0 | 0 | 0 | 0 |
| *Sceloporus grammicus* | 0 | 1a | 0 | 0 | 0 | 0 | 0 | 0 |
| *Sceloporus jarrovi* | 0 | 0 | 0 | 1a | 0 | 0 | 0 | 0 |
| *Phrynosoma orbiculare* | 0 | 1a | 0 | 0 | 0 | 0 | 0 | 0 |
| *Barisia imbricata* | 0 | 1a | 0 | 0 | 0 | 0 | 0 | 0 |
| *Basiliscus sp* | 0 | 0 | 0 | 0 | 1 | 0 | 0 | 0 |
| *Basiliscus vittatus* | 0 | 0 | 0 | 0 | 1 | 0 | 0 | 0 |
| *Iguana iguana* | 0 | 0 | 0 | 0 | 0 | 0 | 0 | 1i |
| *Ctenosaura acanthura* | 0 | 0 | 0 | 0 | 1 | 0 | 0 | 0 |
| *Ctenosaura pectinata* | 0 | 0 | 0 | 0 | 0 | 0 | 1 | 0 |
| *Nerodia erythrogaster* | 0 | 0 | 1 | 0 | 0 | 0 | 0 | 0 |
| *Conopsis lineatus* | 0 | 0 | 0 | 0 | 0 | 1e | 0 | 0 |
| *Leptodeira septentrionalis* | 0 | 0 | 0 | 0 | 0 | 1e | 0 | 0 |
| Anguidae | 0 | 0 | 0 | 0 | 1 | 0 | 0 | 0 |
| **Aves** | 64 | 0 | 1 | 0 | 7 | 0 | 1 | 0 |
| *Columba livia* | 14 | 0 | 0 | 0 | 1 | 0 | 2 | 0 |
| Columbidae | 9 | 0 | 1 | 0 | 0 | 0 | 1 | 0 |
| *Zenaida asiática* | 4 | 1b | 0 | 0 | 3 | 0 | 1 | 1f |
| *Columbina inca* | 6 | 1b | 0 | 0 | 0 | 0 | 0 | 0 |
| Passeriformes | 2 | 0 | 0 | 0 | 0 | 0 | 3 | 0 |
| *Streptopelia decaocto* | 3 | 0 | 0 | 0 | 0 | 0 | 1 | 0 |
| *Quiscalus mexicanus* | 2 | 0 | 0 | 0 | 1 | 0 | 1 | 0 |
| *Zenaida macroura* | 2 | 1b | 0 | 0 | 0 | 0 | 0 | 0 |
| *Bubulcus Ibis* | 0 | 0 | 0 | 0 | 0 | 0 | 3 | 0 |
| *Calidris sp* | 1 | 0 | 1 | 0 | 0 | 0 | 0 | 0 |
| *Melanerpes aurifons* | 2 | 0 | 0 | 0 | 0 | 0 | 0 | 0 |
| *Turdus rufopaliatus* | 1 | 1b | 0 | 0 | 0 | 0 | 0 | 0 |
| *Turdus migratorius* | 0 | 1a, 1b | 0 | 0 | 0 | 0 | 0 | 0 |
| *Passer domesticus* | 2 | 0 | 0 | 0 | 0 | 0 | 0 | 0 |
| *Callipepla gambelii* | 1 | 0 | 0 | 0 | 0 | 0 | 0 | 0 |
| *Cyrtonyx montezumae* | 0 | 1b | 0 | 0 | 0 | 0 | 0 | 0 |
| *Columbina talpacoti* | 1 | 0 | 0 | 0 | 0 | 0 | 0 | 0 |
| Picidae | 0 | 1a | 0 | 0 | 0 | 0 | 0 | 0 |
| *Melanerpes formicivorus* | 0 | 1a | 0 | 0 | 0 | 0 | 0 | 0 |
| *Colaptes auratus* | 0 | 1a | 0 | 0 | 0 | 0 | 0 | 0 |
| *Euptilotis neoxenus* | 0 | 1a | 0 | 0 | 0 | 0 | 0 | 0 |
| *Thryomanes bewickii* | 1 | 0 | 0 | 0 | 0 | 0 | 0 | 0 |
| *Mimus gilvus* | 0 | 0 | 0 | 0 | 1 | 0 | 0 | 0 |
| *Toxostoma sp* | 0 | 1b | 0 | 0 | 0 | 0 | 0 | 0 |
| *Aphelocoma sp* | 0 | 1a | 0 | 0 | 0 | 0 | 0 | 0 |
| *Piranga sp* | 0 | 1a | 0 | 0 | 0 | 0 | 0 | 0 |
| *Piranga ludoviciana* | 0 | 1b | 0 | 0 | 0 | 0 | 0 | 0 |
| *Sialia mexicana* | 0 | 1a | 0 | 0 | 0 | 0 | 0 | 0 |
| *Junco phaoenotus* | 0 | 1a | 0 | 0 | 0 | 0 | 0 | 0 |
| *Pipilo erythrophtalmus* | 0 | 1a | 0 | 0 | 0 | 0 | 0 | 0 |
| *Sturnella lilianae* | 0 | 1b | 0 | 0 | 0 | 0 | 0 | 0 |
| *Spinus sp* | 0 | 1a | 0 | 0 | 0 | 0 | 0 | 0 |
| Total records | 122 | 30 | 106 | 4 | 80 | 5 | 48 | 6 |
