# Supplementary material for: Urban areas promotes shifts in the proportion of prey consumed by four raptor species (Accipitridae) in Mexico
Source: PeerJ. 2025 Oct 29;13:e20307. doi: 10.7717/peerj.20307 (PMC12579481; doi:10.7717/peerj.20307)
Supplement: Supplemental Information 4 — We calculated the distance from each predation record to the nearest human settlement using a geographic information system (ESRI, 2011). The distance of records that occurred within the polygons of human settlements was “0”. These records were classified as urban records, while records that occurred outside the polygons of human settlements were categorized as non-urban [file peerj-13-20307-s004.pdf]

Supplemental Information 3.

Calculation of the distance from each predation record to the nearest human settlement.

We calculated the distance from each predation record to the nearest human settlement using a geographic information system (ESRI 2011). The distance of records that occurred within the polygons of human settlements was "0". These records were classified as urban records, while records that occurred outside the polygons of human settlements were categorized as non-urban.

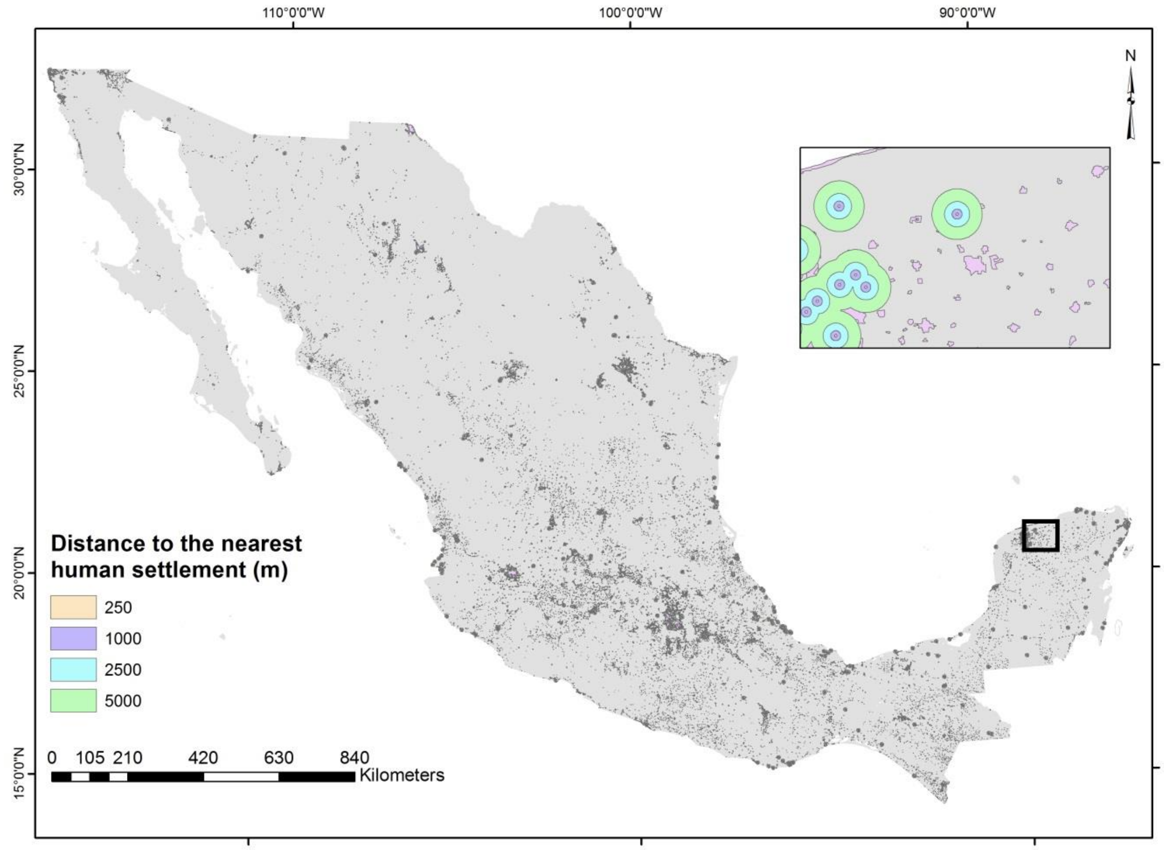

This map of Mexico shows the ring-shaped buffer zones around each raptor predation record. These ring-shaped buffer zones range from 0 to 5000 meters around each predation record. Due to the large spatial scale of the study area (Mexico), a close-up view of how ring-shaped buffer zones are displayed is shown in the upper right corner.

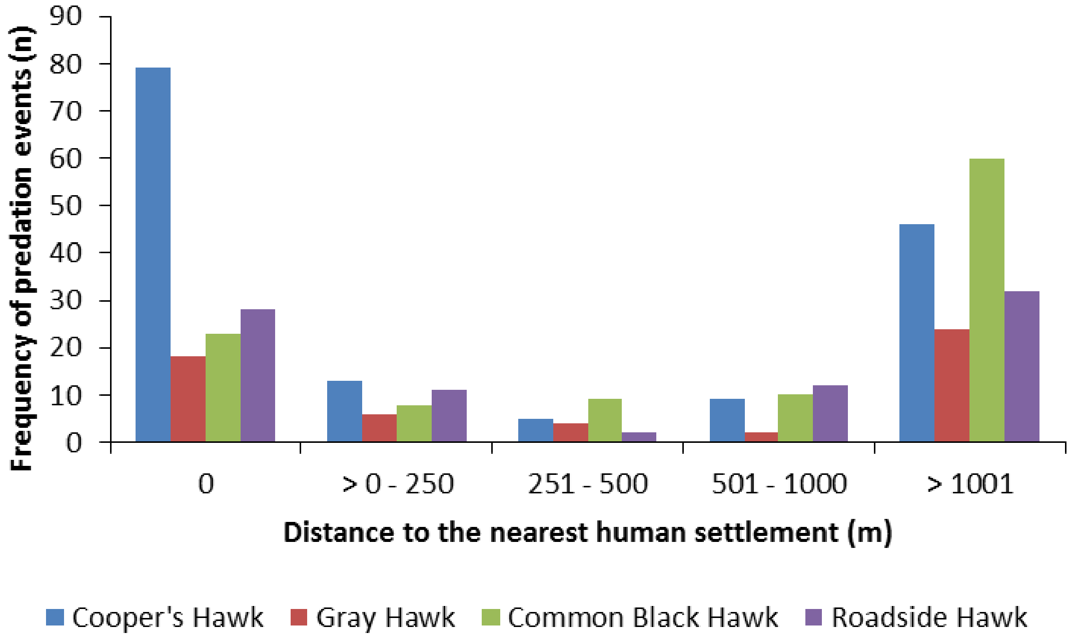

This graph illustrates the frequency of raptor predation records in Mexico and the distance of those records from the nearest human settlement. Most predation records for Gray Hawks, Common Black Hawks, and Roadside Hawks occurred outside urban areas (67%-79%). However, for Cooper's hawks, the frequency of records was similar inside and outside urban areas (52% and 48%, respectively).
